# Validation of the Updated Porto Proposal in Papillary Thyroid Microtumors: Analysis of Cases at a University Hospital in Catalonia, Spain

**DOI:** 10.3390/cancers17122021

**Published:** 2025-06-17

**Authors:** Karmele Saez de Gordoa, Elias Tasso, Alexandre Rei, Martin Ramonda, Belinda Salinas, Sandra Cobo-Lopez, Aida Orois, Amparo Cobo, Marti Manyalich-Blasi, Teresa Ramón y Cajal, Mireia Mora, Felicia Hanzu, Oscar Vidal Pérez, Maria Teresa Rodrigo-Calvo

**Affiliations:** 1 Pathology Department, Centre of Biomedical Diagnosis, Hospital Clínic, 08036 Barcelona, Spain; saezdegord@clinic.cat (K.S.d.G.); tasso@clinic.cat (E.T.); alrey@clinic.cat (A.R.); ramonda@clinic.cat (M.R.); bsalinas@clinic.cat (B.S.); scobo@clinic.cat (S.C.-L.); 2 Institut d’Investigacions Biomèdiques August Pi I Sunyer (IDIBAPS), 08036 Barcelona, Spain; 3 Endocrinology Department, Clinic Institute of Digestive and Metabolic Diseases, Hospital Clínic, 08036 Barcelona, Spain; aorois@clinic.cat (A.O.); mporta@clinic.cat (M.M.); fhanzu@clinic.cat (F.H.); 4 Radiodiagnosis Department, Imaging Diagnostic Center, Hospital Clínic, 08036 Barcelona, Spain; acobo@clinic.cat; 5 Surgery Department, Clinic Institute of Digestive and Metabolic Diseases, Hospital Clínic, 08036 Barcelona, Spain; manyalich@clinic.cat (M.M.-B.); ovidal@clinic.cat (O.V.P.); 6 Oncology Department, Institute of Cancer and Blood Diseases, Hospital Clínic, 08036 Barcelona, Spain; ramonycajal@clinic.cat; 7 Medicine Department, University of Barcelona (UB), 08036 Barcelona, Spain; 8 Surgery and Surgical Specialties Department, University of Barcelona (UB), 08036 Barcelona, Spain

**Keywords:** papillary thyroid microcarcinoma, papillary microtumor, Porto proposal, thyroid cancer, histopathology, risk stratification, minimally invasive treatment

## Abstract

**Background/Objectives**: Given the high incidence and generally favorable prognosis of papillary thyroid microcarcinomas (PTMs), the Porto Proposal aims to refine the management of these tumors. It designates tumors lacking certain risk factors as papillary microtumors (PMTs) to avoid overtreatment and reduce patient stress. The updated Porto Proposal (uPp) suggests criteria for reclassifying incidental PTMs as PMTs. This study seeks to validate these criteria using data from a university hospital in Catalonia, Spain, and assess the clinical and pathological characteristics of PTMs. **Methods**: This retrospective study analyzed patients diagnosed with PTM (≤1 cm) at a university hospital from 2000 to 2024. The study examined variables, including lymph node positivity, incidental diagnosis, tumor location, histological type, treatment, multifocality, age at diagnosis, tumor size, and survival. The uPp criteria were applied to reclassify PTMs into PMTs or PMCs (true papillary microcarcinomas). Student’s *t*-test and chi-square tests were used to evaluate the associations between these variables and the uPp classification. **Results**: The cohort comprised 107 patients, with 77 (72%) women and 30 men. The mean age at diagnosis was 54.5 years. Out of the total, 77 (72%) cases were reclassified as PMTs and 30 (28%) as PMCs according to the uPp criteria. PMC tumors were larger (mean size 4.5 mm vs. 3.3 mm for PMT, *p* = 0.014) and were significantly associated with multifocality (52.2%; *p* = 0.004). Most lymph node-positive cases were classified as PMCs (69.2%; *p* < 0.001) and were multifocal and bilateral more commonly. However, no significant differences in outcomes between PMCs and PMTs were found (*p* = 0.188). Follicular histology was significantly more common in PMTs (87.0%, *p* < 0.001) and rarely had lymph node metastases (4.6%; *p* = 0.047). **Conclusions**: The updated Porto Proposal (uPp) effectively identifies PTMs with minimal malignant potential, distinguishing between PMT and PMC. The findings support the protocol’s use in reducing unnecessary treatments and psychological stress for patients. The study highlights significant clinical and pathological differences between PTM subtypes, reinforcing the protocol’s applicability in daily pathological practice.

## Simple Summary

Papillary thyroid microcarcinomas (PTMs) are small thyroid tumors with generally positive outcomes. However, many patients undergo unnecessary treatments, causing stress and potential complications. The Porto Proposal suggests reclassifying certain low-risk PTMs as papillary microtumors (PMTs) to avoid overtreatment. In this study, we used data from a hospital in Catalonia, Spain, to test the updated Porto Proposal (uPp) criteria, aiming to better identify which tumors need less aggressive management. Our findings indicate that the uPp criteria effectively distinguish between low-risk PMTs and true papillary microcarcinomas (PMCs). This approach could reduce unnecessary treatments and improve patient’s quality of life, providing valuable insights for pathologists and clinicians managing thyroid cancer.

## 1. Introduction

Papillary thyroid microcarcinomas (PTMs) represent a specific subset of papillary thyroid carcinomas, defined by the World Health Organization (WHO) as tumors measuring ≤ 1 cm in maximum diameter [1]. Due to their small size, PTMs are typically asymptomatic and are often discovered incidentally through non-thyroid-specific imaging studies, such as neck MRI, or through histological analysis of thyroid glands removed for other reasons [2,3]. Indeed, a retrospective analysis indicates that PTMs are present in 5% to 10% of thyroid glands removed due to benign conditions or examined in autopsies of patients who died from unrelated causes. In some cases, diagnosis occurs after neck palpation detects a nodule, leading to a thyroid ultrasound, which ultimately identifies the PTM. Additionally, in countries with thyroid cancer screening programs, ultrasound is the most common tool for detecting these tumors [2].

Despite their high incidence, most PTMs exhibit indolent behavior, with extremely low mortality rates and a low propensity for clinical progression [4]. The increased detection of PTMs has led to a re-evaluation of their clinical management, with a growing focus on active surveillance instead of immediate surgery for tumors without aggressive features [5]. However, debates persist regarding risk stratification and the need to define more precise criteria that distinguish low-risk PTMs, which can be managed conservatively, from those with greater potential for progression [6].

In response to this issue, the Porto Proposal (Pp) was introduced in 2003 as an attempt to reclassify certain PTMs without risk factors under the designation papillary microtumors (PMT), avoiding the “carcinoma” connotation and reducing the psychological impact of the diagnosis on patients [7]. More recently, the updated Porto Proposal(uPp) has refined these criteria, establishing clearer restrictions to differentiate PMTs from PTMs with aggressive potential [8]. This approach has been validated in retrospective studies, demonstrating that PMTs lack lymph node metastases, distant metastases, and associated mortality, which supports their conservative management [8,9].

Despite the growing consensus in favor of active surveillance, certain histological subtypes of PTMs, such as the tall cell variant (TCV), exhibit more aggressive behavior and may justify a more interventionist management [10]. Furthermore, while the presence of mutations such as BRAFV600E has been historically associated with an increased risk of invasion and recurrence, recent studies suggest that BRAF mutation alone does not independently predict poor outcomes. Rather, the coexistence of BRAFV600E with TERT promoter mutations appears to confer a higher risk of disease progression, supporting the potential role of combined molecular biomarkers in clinical decision-making [11,12,13].

This study aims to evaluate the applicability of the updated Porto Proposal (uPp) criteria in a cohort of patients from a university hospital in Catalonia, Spain, analyzing the clinical and pathological characteristics of PTMs to determine the validity and utility of this classification scheme in diagnostic and therapeutic practice.

## 2. Materials and Methods

### 2.1. Study Design

A retrospective observational study was conducted to evaluate and compare the clinical and pathological characteristics of papillary thyroid microcarcinomas. This study analyzed cases diagnosed between 2000 and 2024 in our institution. The primary objective was to identify differences and similarities between these two entities to enhance their management and prognostic assessment. The study included patients who met strict diagnostic criteria, excluding cases with insufficient information or ambiguous classification.

### 2.2. Patient Selection

The study population consisted of patients with a confirmed pathological diagnosis of papillary thyroid microcarcinomas (PTMs), defined as papillary thyroid carcinomas measuring ≤ 10 mm in maximum dimension. The patients were classified according to the updated Porto Proposal criteria into papillary microcarcinomas (PMCs) and incidental papillary microtumors (PMTs) [8]. Papillary thyroid microtumors (PMTs) were defined based on the following criteria:

(a) patients ≥18 years;
(b) without a known family history of thyroid cancer;
(c) for tumors detected incidentally;
(d) measuring less than 1 cm (in the case of multiple PTMs the sum of their diameters does not exceed 1 cm);
(e) lacking worrisome features.

On the contrary, cases with high-risk features (including microscopic extrathyroidal extension, angioinvasion, tall cell subtype, solid growth pattern, or hobnail cells) or not fulfilling the definition of PMTs were classified as PMCs. Patients with a prior history of thyroid carcinoma were excluded from the study, as well as those with incomplete or unclear clinical or pathological data.

### 2.3. Patient Selection Process

**Figure 1:**
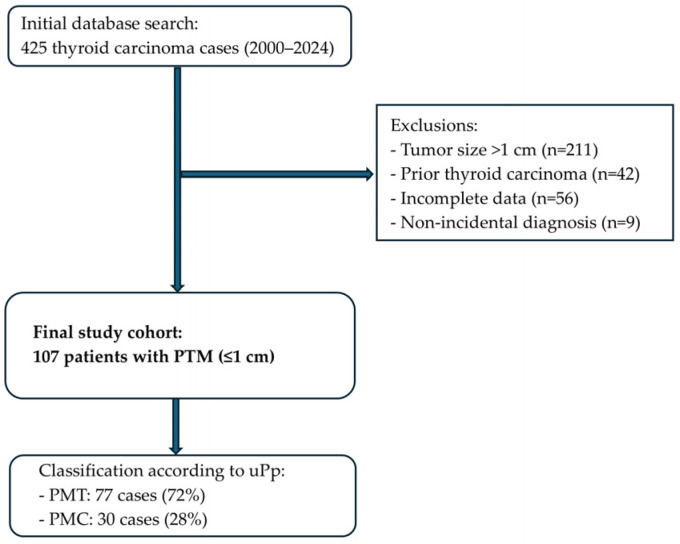
Flowchart summarizing the selection process for the study cohort. From an initial database of 425 thyroid carcinoma cases diagnosed between 2000 and 2024, exclusions were applied for tumor size > 1 cm, prior thyroid carcinoma history, incomplete clinical or pathological data, and non-incidental diagnosis. The final study cohort comprised 107 patients with papillary thyroid microcarcinomas (PTMs ≤ 1 cm), subsequently classified according to the updated Porto Proposal criteria into papillary microtumors (PMTs) and papillary microcarcinomas (PMCs).

We reviewed 425 cases of thyroid carcinoma diagnosed between 2000 and 2024 at our institution. Cases were excluded if they presented a tumor size larger than 1 cm (n = 211), a history of previous thyroid carcinoma (n = 42), incomplete clinical or pathological data (n = 56), or a non-incidental diagnosis (n = 9). After applying these criteria, 107 patients were included in the study cohort (Figure 1).

### 2.4. Data Collection

Data were extracted systematically from electronic medical records, histopathology reports, and imaging studies. Demographic data, including age and sex, were collected along with detailed information about the tumor, such as its size, location within the thyroid gland, histological subtype, presence of capsular invasion, and extrathyroidal extension. Additional variables included the lymph node involvement, specifying the number, size, and location of metastatic lymph nodes when applicable. The multifocality of the tumor, the surgical interventions performed (e.g., lobectomy or total thyroidectomy), and the postoperative treatments, including radioactive iodine therapy, were recorded. Clinical outcomes such as recurrence, survival, and thyroid-related mortality were also documented.

### 2.5. Statistical Analysis

Continuous variables were summarized as means and standard deviations, while categorical variables were expressed as counts and percentages. For comparisons between groups, a Student’s *t*-test was used for continuous variables. For categorical variables, Fisher’s exact test was applied when the expected frequency in any cell was less than five, in order to maintain statistical accuracy in small subgroups. A *p*-value < 0.05 was considered statistically significant. Statistical analyses were performed using R statistical software (R version R 4.4.2, R Foundation for Statistical Computing, Vienna, Austria. https://www.R-project.org/ accessed on 6 February 2025).

### 2.6. Ethical Considerations

This study received approval from the Ethics Committee of the Hospital Clínic of Barcelona and was conducted in accordance with the principles outlined in the Declaration of Helsinki. Since the study was retrospective and based on existing clinical data, the requirement for individual informed consent was waived by the Ethics Committee. To ensure confidentiality, all patient data were anonymized during the process of extraction and analysis. Identifiable information was replaced with unique study codes, and all analyses were conducted with strict adherence to data protection regulations.

## 3. Results

### 3.1. Clinicopathological Characteristics of PTMs and Reclassification

The cohort consisted of 107 patients, predominantly female (72%), with a mean age at diagnosis of 54.5 years. In total, 77 cases (71.96%) were reclassified as papillary microtumors (PMTs), while 30 cases (28.04%) were classified as papillary microcarcinomas (PMCs) according to the uPp criteria. PMCs were identified through clinical suspicion or targeted radiological investigations, often prompted by palpable nodules, symptoms, or imaging findings suggestive of thyroid pathology. PMTs, in contrast, were incidentally discovered during imaging studies performed for unrelated reasons, such as investigations for cervical pain or vascular assessments, or were identified in a thyroidectomy specimen for a benign diagnosis.

We compared the clinical and pathological features of PMCs and PMTs and did not observe any differences between the sexes or locations of the tumor. Interestingly, multifocality was more common in PMCs than in PMTs. We observed significant differences regarding tumor subtype, as follicular histology was far more common in PMT cases (84.6%, *p* < 0.001), while all tall cell cases were classified as PMCs. The tall cell variant was observed in seven cases, representing 6.5% of the cohort. All tall cell variant cases were classified as PMCs according to the updated Porto Proposal criteria. Additionally, PMC tumors were larger, with a mean size of 4.5 mm, in contrast to 3.3 mm for PMTs (*p* = 0.015). The age of diagnosis did not vary significantly between PMC and PMT cases, although PMCs tended to appear at a younger age (Table 1, Figure 1).

### 3.2. Lymph Node Metastasis and Outcomes in Patients

**Figure 2:**
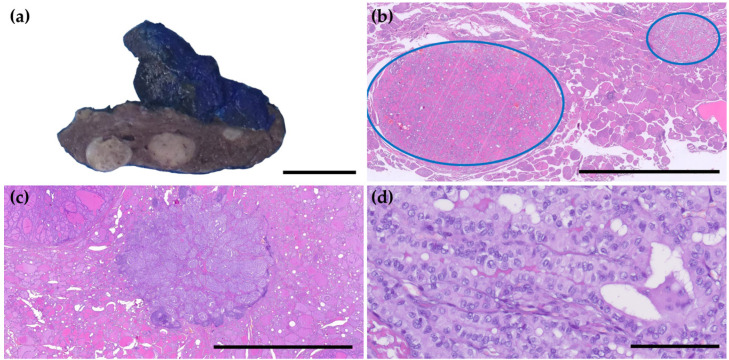
Features associated with papillary microcarcinomas. (**a**) Macroscopic image of a multifocal tumor (black line: 1 cm); (**b**) Multifocal tumor, with two foci of PTMs delineated in blue (1× magnification, black line 5 mm); (**c**) Tall cell subtype of PTMs measuring 6 mm, classified as a PMC (1× magnification, black line 5 mm); (**d**) High-power view of the tall cell tumor, showing a trabecular pattern, with cell height at least 2 times the cell width and distinct cell borders (40× magnification, black line 0.1 mm).

**Figure 3:**
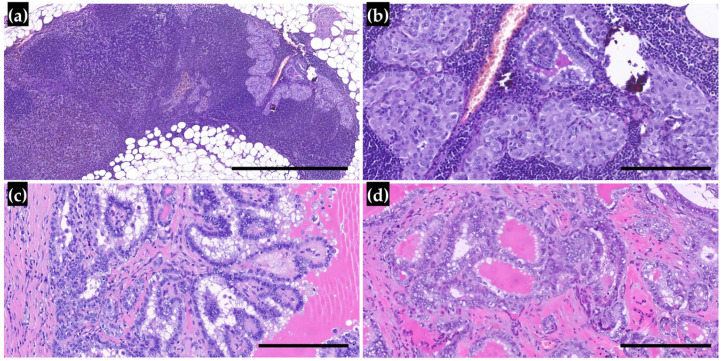
(**a**,**b**) Cervical lymph node with a lymph node metastasis of papillary thyroid carcinoma (5× and 20× magnification, black line: 1 mm and 0.2 mm, respectively); (**c**) Classic subtype of PTM, showing true papillae lined by cells with irregular and clear nuclei (20× magnification, black line 0.2 mm); (**d**) PTM with a follicular growth pattern, which is associated with a low risk of LNMs (20× magnification, black line 0.2 mm).

In our cohort, 13 patients (12.1%) had lymph node metastases (LNMs), and those were significantly more frequent in cases classified as PMCs (69.2%). LNMs were associated with multifocal tumors and, subsequently, with tumors located in both lobes. Interestingly, LNMs were associated with the histologic subtype, but the tall cell subtype did not confer a higher risk of spreading to lymph nodes than the classic subtype. Lymph node involvement was observed in patients diagnosed at a younger age and with larger tumor sizes, but these differences were not statistically significant (Table 2, Figure 2 and Figure 3).

The mean follow-up time was 36.64 months, and one patient classified as PMC presented recurrence, with cervical LNM identified 19 months after the initial surgery. Two patients with a PMT died due to causes unrelated to the thyroid pathology, whereas none of the PMC patients were deceased by the end of follow-up. The different outcomes described in PMC and PMT patients were not statistically significant (*p*-value = 0.1876).

## 4. Discussion

The findings from our study on papillary thyroid microcarcinomas (PTMs) provide critical insights into the clinical and pathological features of these tumors. Given the fact that the incidence of thyroid cancer has significantly increased [14], mostly due to PTMs, it is critical to redefine this entity. Moreover, the increased population aging will probably lead to a rise in this diagnosis, as these tumors are more often detected in older patients [15,16].

Seeking to distinguish a PTM subgroup with a better prognosis, we reclassified this tumor according to the updated Porto Proposal (uPp) criteria. Notably, most cases were redefined as PMTs, and only 28% of cases were true PMCs. This distribution underscores the proposal’s emphasis on identifying low-risk tumors that may not require aggressive treatment, thus aligning with the growing consensus that many PTMs exhibit indolent behavior and favorable outcomes [17,18]. Nonetheless, a subset of patients with PMCs exhibit characteristics that warrant closer monitoring and potentially more aggressive treatment [8,19].

PTMs are characterized by their indolent clinical behavior and slow growth. However, cervical lymph node metastasis (LNM) has been found in up to 24–64% of patients with PTMs [20]. LNM is strongly associated with recurrence in papillary thyroid carcinoma [19,21]. In our cohort, 69.2% of lymph node-positive cases were classified as PMCs, further emphasizing the need for careful risk stratification in this population. However, four patients who were reclassified as having PMTs had LNM, even though their cancer did not recur afterward. This contradicts the findings of Aliyev et al., who did not find any LNM in their PMT cases [8]. This highlights the high variability in PMTs; while the overall prognosis for PMTs is generally positive, some cases may have worse outcomes [17,22].

The size differences observed between PMC and PMT patients are particularly noteworthy. PMC patients presented with larger tumors, which aligns with the existing literature suggesting that larger tumor sizes are associated with more aggressive disease features [23]. Moreover, previous studies indicate that larger tumors and younger patients are more likely to exhibit lymph node metastasis (LNM) [24]. In our cohort, patients harboring LNM tended to have larger tumors and be younger; however, these results were not significant. Although the difference in mean tumor size between groups (3.3 mm vs. 4.5 mm) may appear small and is unlikely to impact disease-specific survival, it could still be clinically relevant. Slightly larger microcarcinomas have been associated with higher rates of multifocality and lymph node metastases—factors that can influence surgical planning and postoperative surveillance strategies. Therefore, even subtle size distinctions may contribute to a more personalized risk assessment and management approach.

Our findings indicate that multifocal tumors do not meet the criteria of PMTs more frequently, and we confirm the results of previous studies that have identified multifocality as a risk factor for LNM in PTMs [23]. The potential association of multifocal tumors with more aggressive behavior highlights the importance of thorough preoperative evaluation and may indicate a more aggressive surgical intervention in these cases. The involvement of both thyroid lobes by the PTMs was associated with LNM in our cohort. This result, however, was not confirmed in a recent meta-analysis [19,25].

The histological findings in our study also provide valuable insights into the biological behavior of PTMs. The predominance of follicular histology in PMT cases (84.6%) suggests that histological subtypes may serve as an important factor in determining the aggressiveness of the tumor [1]. The tall cell subtype, known for its association with poorer outcomes, necessitates vigilant monitoring and may influence treatment decisions [10]. This aligns with the findings of other studies that have highlighted the importance of histological features in risk stratification for PTMs [8]. Surprisingly, we found that LNM was more common in the classic subtype than the tall cell variant, which is not an expected finding [23].

Interestingly, our study found no significant differences in survival between PMC and PMT patients, and none of the patients died of a cause related to thyroid cancer. This finding suggests that even among patients classified as PMC, the overall prognosis remains favorable, which is consistent with the notion that most PTMs are indolent and may not necessitate aggressive treatment [23,26]. The excellent long-term survival rates associated with this tumor reinforce the argument for a more conservative management approach [5,9,23,27]. Considering the risks associated with thyroidectomy, including vocal cord paralysis, hypoparathyroidism, and the requirement for levothyroxine replacement therapy, treatment strategies have evolved towards more conservative approaches. Thyroid lobectomy and active surveillance have proven to be safe and effective alternative therapeutic options in selected cases, with favorable long-term clinical outcomes [19,28,29]. For that matter, the updated American Thyroid Association (ATA) guidelines advocate for active surveillance in select cases of papillary microcarcinoma, particularly for patients without aggressive features or significant comorbidities [30]. Furthermore, the adoption of these less invasive approaches has launched a debate on the decision-making process in the management of PTMs, as well as its impact on aspects such as the patient’s quality of life and the impact on healthcare costs [19,31]. However, despite new evidence and updated guidelines, in many institutions total thyroidectomy and the use of radioiodine (RAI) remain widely applied treatments, indicating that the treatment of PTMs remains predominantly aggressive [32,33].

Additionally, the ATA has proposed changes in the management of thyroid nodules that could influence future thyroid cancer incidence [2,27]. According to these recommendations, thyroid nodules measuring 1 cm or less should not undergo biopsy unless they exhibit clinical or ultrasound features indicative of aggressive malignancy, such as clear extrathyroidal extension or vocal cord paralysis [34]. Since PMTs do not display these characteristics, it is likely that a larger number of small nodules will not be evaluated through biopsy, potentially reducing the reported incidence of thyroid cancer. While there is no definitive evidence yet on the impact of these recommendations, some recent studies suggest that the annual thyroid cancer diagnosis rate has started to decline [28,30].

One of the limitations of our study is related to the patient selection process. Several cases were excluded due to incomplete clinical or pathological data, a prior history of thyroid carcinoma, or a diagnosis based on clinical suspicion rather than incidental findings. Although these criteria were necessary to ensure the rigorous application of the updated Porto Proposal (uPp) classification, they could introduce selection bias. As a result, our cohort may not fully represent the entire spectrum of papillary thyroid microcarcinomas diagnosed in routine clinical practice. Future prospective studies, including larger and unselected populations, will be needed to further validate these findings. Another limitation of our study is the absence of a control group consisting of benign thyroid tumors. Including such a comparison group could enhance understanding of the biological behavior differences and further validate the applicability of the updated Porto Proposal criteria. Future studies addressing this aspect are warranted. Additionally, the relatively short median follow-up period (36 months) can be explained by the significant increase in incidental detection of thyroid microcarcinomas in recent years due to the expanded use of neck imaging studies.

Although the updated Porto Proposal criteria allow for more refined risk stratification, the presence of lymph node metastases in a small subset of PMT cases in our cohort highlights the intrinsic biological variability of papillary thyroid microtumors. Therefore, thorough clinical follow-up remains essential, even for tumors initially classified as low risk.

The application of the updated Porto Proposal provides a practical and reproducible framework for distinguishing low-risk PTMs. Its widespread adoption could promote conservative management strategies, reduce unnecessary surgeries, and improve the quality of life for patients across different healthcare settings in Europe and beyond.

## 5. Conclusions

In conclusion, our study contributes to the evolving understanding of papillary thyroid microcarcinomas and the implications of the Porto Proposal for clinical practice. The reclassification of PTMs based on the uPp criteria allows for a more tailored approach to management, emphasizing the need for careful evaluation of tumor characteristics and patient demographics. While the majority of PTMs may be safely monitored, a proactive search for high-risk features is required to identify PMC patients who will require a more aggressive approach to treatment. Future research should focus on long-term outcomes associated with different management strategies to further refine the guidelines for the management of papillary thyroid microcarcinomas.

## Figures and Tables

**Table 1:** Clinical and pathologic characteristics of patients with PMCs and PMTs.

		PMC N (%)	PMT N (%)	*p*-Value
Sex	Male	9 (30)	21 (70)	0.7779
	Female	21 (27.3)	56 (72.7)	
Location	Right lobe	15 (31.9)	32 (68.1)	0.2079
	Left lobe	9 (20.5)	35 (79.5)	
	Isthmus	2 (25)	6 (75)	
	Both lobes	4 (57.1)	3 (42.9)	
Focality	Unifocal	18 (21.7)	65 (78.3)	0.0041
	Multifocal	12 (52.2)	11 (47.8)	
Subtype	Classic	10 (35.7)	18 (64.3)	<0.0001
	Follicular	10 (15.4)	55 (84.6)	
	Tall cell	7 (100)	0 (0)	
	Oncocytic	1 (25)	3 (75)	
	Solid	1 (50)	1 (50)	
	Warthin-like	1 (100)	0 (0)	
Treatment	Right HT	5 (29.4)	12 (70.6)	0.5813
	Left HT	3 (17.6)	14 (82.4)	
	TT	22 (30.1)	51 (69.9)	
		**Mean**	**Mean**	
Age at diagnosis		50.23	56.12	0.0811
Size (mm)		4.53	3.26	0.0147

Abbreviations: HT, hemithyroidectomy; PMT: papillary microtumor; PMC: papillary microcarcinoma; TT, total thyroidectomy.

**Table 2:** Features of patients with and without lymph node metastases (LNMs).

		LNM N (%)	No LNM N (%)	*p*-Value
PMC/PMT	PMC	9 (30)	21 (70)	0.0004
	PMT	4 (5.2)	73 (94.8)	
Sex	Male	3 (10)	27 (90)	0.671
	Female	10 (13)	67 (87)	
Location	Right lobe	4 (8.5)	43 (91.5)	0.0027
	Left lobe	4 (9.1)	40 (90.9)	
	Isthmus	1 (12.5)	7 (87.5)	
	Both lobes	4 (57.1)	3 (42.9)	
Focality	Unifocal	5 (6)	78 (94)	0.0002
	Multifocal	8 (34.8)	15 (65.2)	
Incidental finding	Yes	3 (4.2)	68 (95.8)	0.0004
	No	10 (27.8)	26 (72.2)	
Subtype	Classic	7 (25)	21 (75)	0.0465
	Follicular	3 (4.6)	62 (95.4)	
	Tall cell	1 (14.3)	6 (85.7)	
	Oncocytic	1 (25)	3 (75)	
	Solid	1 (50)	1 (50)	
	Warthin-like	0 (0)	1 (100)	
Treatment	Right HT	1 (5.9)	16 (94.1)	0.3997
	Left HT	1 (5.9)	16 (94.1)	
	TT	11 (15.1)	62 (84.9)	
		**Mean**	**Mean**	
Age at diagnosis		47	55.5	0.0807
Size (mm)		4.62	3.48	0.1188

Abbreviations: HT, hemithyroidectomy; PMT: papillary microtumor; PMC: papillary microcarcinoma; TT, total thyroidectomy.

## Data Availability

The datasets generated and/or analyzed during the current study are available from the corresponding author upon reasonable request.

## Abbreviations

The following abbreviations are used in this manuscript:

PTM	Papillary Thyroid Microcarcinoma
PMT	Papillary Microtumor
PMC	Papillary Microcarcinoma
